# Imaging Arm Regeneration: Label-Free Multiphoton Microscopy to Dissect the Process in *Octopus vulgaris*

**DOI:** 10.3389/fcell.2022.814746

**Published:** 2022-02-04

**Authors:** Pamela Imperadore, Roberta Galli, Martin J. Winterhalder, Andreas Zumbusch, Ortrud Uckermann

**Affiliations:** ^1^ Department of Biology and Evolution of Marine Organisms, Napoli, Italy; ^2^ Association for Cephalopod Research—CephRes, Napoli, Italy; ^3^ Clinical Sensoring and Monitoring, Anesthesiology and Intensive Care Medicine, TU Dresden, Dresden, Germany; ^4^ Medical Physics and Biomedical Engineering, Faculty of Medicine Carl Gustav Carus, TU Dresden, Dresden, Germany; ^5^ Department of Chemistry, University of Konstanz, Konstanz, Germany; ^6^ Department of Neurosurgery, University Hospital Carl Gustav Carus and Faculty of Medicine, TU Dresden, Dresden, Germany; ^7^ Division of Medical Biology, Department of Psychiatry and Psychotherapy, University Hospital Carl Gustav Carus and Faculty of Medicine, TU Dresden, Dresden, Germany

**Keywords:** spontaneous functional regeneration, vibrational spectroscopy, label-free imaging, cephalopod mollusks, hemocytes, chromatophores

## Abstract

Cephalopod mollusks are endowed with an impressive range of features that have captured the attention of scientists from different fields, the imaginations of artists, and the interests of the public. The ability to spontaneously regrow lost or damaged structures quickly and functionally is among one of the most notable peculiarities that cephalopods possess. Microscopical imaging techniques represent useful tools for investigating the regenerative processes in several species, from invertebrates to mammals. However, these techniques have had limited use in cephalopods mainly due to the paucity of specific and commercially available markers. In addition, the commonly used immunohistochemical staining methods provide data that are specific to the antigens studied. New microscopical methods were recently applied to vertebrates to investigate regenerative events. Among them, multiphoton microscopy appears promising. For instance, it does not depend on species-related epitopes, taking advantage of the specific characteristics of tissues and allowing for its use in a species-independent way. Here, we illustrate the results obtained by applying this label-free imaging technique to the injured arm of *Octopus vulgaris*, a complex structure often subject to injury in the wild. This approach allowed for the characterization of the entire tissue arm architecture (muscular layers, nerve component, connective tissues, etc.) and elements usually hardly detectable (such as vessels, hemocytes, and chromatophores). More importantly, it also provided morpho-chemical information which helped decipher the regenerative phases after damage, from healing to complete arm regrowth, thereby appearing promising for regenerative studies in cephalopods and other non-model species.

## Introduction

Arm and tentacles in cephalopod mollusks are structures lacking fluid-filled cavities and hard skeletal support. These animals utilize their appendages for environmental exploration, prey manipulation, mating, and communication (for a review, see, for example, Villanueva et al., 2017). *O. vulgaris* arms have been subject to particularly detailed investigation because their peculiar architecture empowers possessing animals with high degrees of freedom in movement, including fine manipulation abilities and muscular softening–stiffening control. The architecture of *O. vulgaris* arms, in turn, has inspired the construction of robotic models (Cianchetti et al., 2011) for medical applications (e.g., minimally invasive surgical systems; Cianchetti et al., 2014) and underwater exploration and sampling (Calisti et al., 2015). Such an extensive use of appendages makes these structures susceptible to a high risk of damage.

It has been estimated that, in *Octopus digueti*, around 26% of the population presents an arm injury (Voight, 1992), an incidence reaching 51% in *O. vulgaris* (Florini et al., 2011). A similar frequency was recently also confirmed by Voss and Mehta (2021), who reported a 59.8% of incidence of injury in one or more arms in museum specimens of various octopus species (i.e., *O. bimaculatus*, *O. bimaculoides*, and *O. rubescens*).

Moreover, the capacity to quickly heal and regenerate these structures, even after severe injury or complete loss, is a peculiar feature of octopuses that has been under investigation since scientists first reported it in 1856 (Steenstrup, 1856).

The majority of studies examining the regenerative capacities of appendages in cephalopods are, however, mostly descriptive and focused on macroscopical events; only in recent years has attention to the cellular and biological machinery of regeneration begun to escalate (Fossati et al., 2013; Fossati et al., 2015; Zullo et al., 2017). One of the main issues hindering an in-depth examination of regenerative processes remains the limited number of markers that are commercially available and specifically designed for these organisms (Wollesen et al., 2009; Imperadore et al., 2018; Zullo et al., 2020), thereby reducing the potential for direct imaging.

Recently, new microscopical methods have been applied to vertebrate models and may help resolve the issue of marker paucity in cephalopods and other non-model invertebrates. Vibrational spectroscopy, the collective term used to describe the analytical techniques of infrared and Raman spectroscopy, appears to be extremely helpful in this sense. Vibrational spectroscopy is a label-free technique for probing vibrational energy levels associated with chemical bonds in a non-destructive and non-invasive manner, allowing the collection of comprehensive information about sample composition. In turn, coherent anti-Stokes Raman scattering (CARS) microscopy is a non-linear variant of Raman spectroscopy that provides intensity information about single-molecular vibration modes at sub-micrometer resolution. In combination with endogenous two-photon excited fluorescence (TPEF) and second harmonic generation (SHG), CARS generates large datasets about the tissue under examination and allows for the acquisition of morpho-chemical information comparable to standard histopathology; for instance, it has been proven suitable both for *ex vivo* and *in vivo* samples (Evans et al., 2005; Bocklitz et al., 2020). One of the potentially most useful applications of this technique is in the evaluation of mammalian disease states, including the production of high-resolution imaging of myelin sheets in physiological and pathological conditions (Huff and Cheng, 2007), and the identification of vessel tissue components to monitor the onset and progression of arterial diseases, such as atherosclerosis or aneurysms (Wang et al., 2008; Sehm et al., 2020). This approach has proved to be exceptionally versatile; it has been applied to the study of axon regeneration after spinal cord or peripheral nerve lesions in mammals (Morisaki et al., 2013), amphibians (Uckermann et al., 2019) and even invertebrates (Imperadore et al., 2018), facilitating comparison among animal species because it does not rely on species-related epitopes.

Recently, we applied CARS microscopy in combination with TPEF and SHG on cephalopods for the first time, using the regenerating pallial nerve of *O. vulgaris* as case study. We highlighted structures, tissues, and cells implicated in regeneration and degeneration by evaluating the status of axons and cells involved in debris removal as well as the connective tissues driving neural fibers. Such evidence would, otherwise, have proven hardly detectable with classical staining methods; at the very least, they would have required several techniques in order to be revealed (Imperadore et al., 2018).

In the current study, we applied multiphoton microscopy to the octopus’ arm, a structure with a high level of structural complexity. The arm is composed of nervous, muscular, endothelial, vascular, and other tissues that, after severe damage or complete loss, regenerate, resuming full functionality and complexity of the uninjured arm. By comparing multiphoton microscopy images with classical histological staining and immunohistochemistry (IHC), we highlighted phases and key events during stump healing and regeneration and detected tissues and cells involved.

## Materials and Methods

### Ethical Statement

Cephalopods are included in the Directive 2010/63/EU and, thus, regulated for their use in scientific research (Fiorito et al., 2014; Fiorito et al., 2015). Experiments included in this study were carried out in 2018 on tissue samples originating from wild animals. This study has been granted an ethical clearance for “label-free multiphoton microscopy for the investigation of the process of arm regeneration in *Octopus vulgaris*” by the institutional AWB (OBA: case 4/2021/ec AWB-SZN -28 June 2021).

### Animals, Surgery, and Sample Collection

This study was carried out on recently deceased *Octopus vulgaris* (*N* = 6; four males, two females, body weight: 194–402 g) collected from fishermen (Bay of Naples, Mediterranean Sea, Italy) during spring (seawater temperature range: 15–20°C). The animals were selected for the presence of one or more damaged arms in the phase of healing or regeneration (following stages reviewed in the study by Imperadore and Fiorito (2018)).

In the cases of octopuses still showing signs of life, the animals were euthanized (3.5% MgCl_2_ in seawater, > 30 min), and death confirmed by transection of the dorsal aorta (Fiorito et al., 2015).

Quality of tissues was assessed through classical histological methods and was found suitable for immunohistochemistry and multiphoton microscopy imaging.

Damaged and corresponding contralateral uninjured arms (control) were harvested (∼3 cm in length) for a total number of 23 samples. For the control, the arm tip (i.e., the most distal part) and a piece of arm at around 50% of its length (proximal) were also collected.

The dissected samples were immediately processed, following the study by Imperadore et al. (2017). In brief, the tissues were fixed in 4% PFA in seawater (3 h), followed by PBS (pH 7.4) washes, and immersion in sucrose 30% (in PBS) until sinking. The samples were then embedded in freezing and blocking medium (OCT; Leica Biosystems) and stored at −80°C until use. Cryostat (Leica CM3050 S) sections (either 30 or 150 µm) were mounted on SuperFrost Plus glass slides.

Two additional control arm tips were harvested, fixed, and stored in PBS to image the whole mount sample.

### Multiphoton Microscopy

The cryostat sections were air-dried for 30 min, rehydrated in PBS, and covered with a glass coverslip. Imaging was performed with an optical microscope Axio Examiner Z.1 coupled to a laser scanning module LSM 7 (Carl Zeiss AG, Jena, Germany) equipped with non-descanned detectors. An erbium fiber laser (Femto Fiber pro NIR from Toptica Photonics AG, Munich, Germany) provides excitation for TPEF and SHG by emitting at 781 nm with a pulse length of 1.2 ps and a maximum nominal power of 100 mW. The TPEF signal was acquired in the spectral range 500–550 nm, while the SHG signal was retrieved using a band-pass filter centered at 390 nm. CARS excitation needed a second laser source (i.e., the Femto Fiber pro TNIR from Toptica Photonics AG) which is tunable in the range 850–1,100 nm and has a pulse length of 0.8 ps. In all CARS experiments, the wavelength was set to 1,005 nm (emitted power 1.5 mW) in order to resonantly excite the symmetric stretching vibration of methylene groups at 2,850 cm^−1^. CARS, TPEF, and SHG were simultaneously excited and acquired with a W Plan-Apochromat 20x/1.0 (Carl Zeiss AG) (for a schematic diagram of the system used for multiphoton microscopy, see Supplementary Figure S1).

For multimodal imaging of thicker slices (150 µm thickness) and whole arm tips, a Leica SP8 CARS microscope with SRS upgrade (special part request, Leica Microsystems GmbH, Mannheim, Germany) was used. A picoEmerald S Optical Parametric Oscillator (APE Angewandte Physik und Elektronik GmbH, Berlin, Germany) provides a Stokes beam at 1,031 nm and a tunable pump beam in the range of 720–970 nm. The two pulse trains (pulse duration 1–2 ps) were spatially and temporally overlapped. The images were acquired using a ×25 water objective (HCX IRAPO L ×25/NA 0.95/water, Leica Microsystems, Mannheim, Germany), and signals in the forward direction were collected using an air condenser (NA 0.4, Leica Microsystems, Mannheim, Germany). Forward CARS (2850cm^−1^, CH_2_-stretch vibration) was spectrally filtered by a short-pass filter SP750, a beam splitter BS560, and a band-pass filter BP670/125. SHG was detected in parallel and spectrally filtered by a short-pass filter SP750, a beam splitter BS560, and a band-pass filter BP465/170. Signals in the epi-direction were spectrally separated in SHG in the range from 400 to 510 nm and TPEF from 515 to 640 nm. All z-stacks were recorded with a voxel size of 0.2 µm × 0.2 µm × 3.0 µm. Z-stacks range from 100 to 130 µm in height.

The resulting multimodal RGB images are represented as follows: red channel = CARS, green channel = TPEF, and blue channel = SHG.

The images were processed with LAS X (Leica Microsystems, Mannheim, Germany) and Zen Blue Edition (Carl Zeiss, AG, Jena, Germany) software.

### Light Microscopy

Following multiphoton imaging, the coverslip was carefully removed in PBS and slides used for immunohistochemistry or stained with hematoxylin and eosin (H&E). The H&E staining protocol consisted of a 2-min bath in Meyer hematoxylin followed by a 5-min step in tap water and 20 s in eosin.

IHC was performed as previously described (Imperadore et al., 2017). In brief, after blocking in normal goat serum (5% NGS, in PBT: PBS Tween 0.1%) for 1 h in RT, the sections were incubated overnight with primary antibody (i.e., anti–acetylated tubulin, SIGMA T6793, dilution 1:1,000; anti–phospho-Histone H3, Sigma H9908, dilution 1:600) in PBT and NGS 1% at 4°C. Following washes in PBT, the sections were incubated with secondary antibodies [1:250, Alexa Fluor goat anti-mouse IgG (H + L) 488 and Alexa Fluor goat anti-rat IgG (H + L) 594] for 1 h at room temperature. DAPI (14.3 μmol L^−1^ in PBT) was used after IHC or on unstained sections to counterstain nuclei.

The sections following IHC protocol were mounted in PBS and imaged again for multiphoton microscopy; H&E sections were, instead, dehydrated in an ethanol series, cleared in xylene, coverslipped using DePex, and imaged using either Axio Examiner Z.1 (Carl Zeiss AG) equipped with the camera AxioCam or Axio Scope A1 (Carl Zeiss AG) equipped with the camera Canon DS126231. The images were processed with Zen Blue Edition (Carl Zeiss, AG, Jena, Germany) software.

## Results

Octopus appendages have sophisticated architecture. The major structures of focus in this study are as follows: 1) the skin, covering the arm (as well as the entire animal’s body; Packard, 1988); 2) the intrinsic musculature, comprising a three-muscular bundle (oblique, longitudinal, and transverse) (for a review, see Kier, 1988) arranged around a 3) central nerve cord, running longitudinally along the entire arm and connecting centrally to the sub-esophageal mass (in the brain) (Graziadei, 1971).

CARS, TPEF, and SHG during multimodal multiphoton imaging on rehydrated cryosections and arm tip whole mounts revealed the architecture of the intact octopus’ appendage, highlighting the entire tissue composition. Injured and healing arms were also imaged, allowing for the identification of main phases of regeneration, including at the levels of cells and tissues.

### Control Uninjured Arm

**The skin.** *Octopus* skin contains various organs and elements (i.e., chromatophores, iridophores, leucophores, and papillae) that can be finely controlled to change the animal’s skin tone and texture, thereby providing the animals with extraordinary camouflaging and interspecific communication (Borrelli et al., 2006; How et al., 2017; for a review, see Packard and Hochberg, 1977). Chromatophores, in particular, are sacculus organs responsible for color change, owing to the presence of diverse pigment granules controlled by muscle bundles that are radially organized to open and close the sacculus (Messenger, 2001).

**FIGURE 1:**
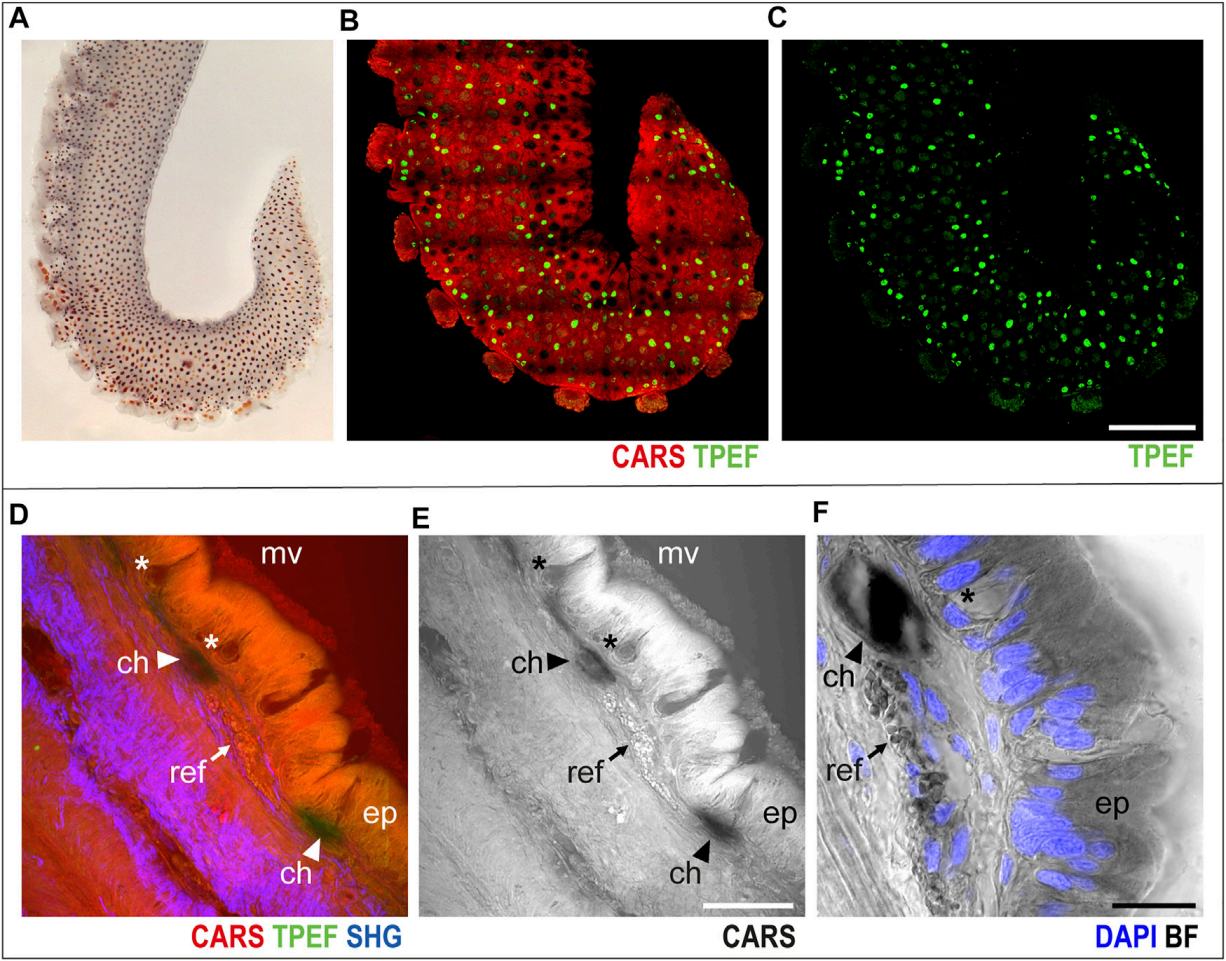
Arm skin structure and reflective elements. An isolated *ex vivo* arm tip imaged before fixation **(A)** with visible chromatophores. The same sample was imaged in whole mount through multiphoton microscopy. **(B)** Entire arm structure is shown in CARS (red), while chromatophores appear in TPEF (green spots in **B,C**). **(D,E)** Imaging of thin sections (30 μm, sagittal plane) highlighted the presence of microvilli (mv) covering the epidermis (ep); mucous cells (asterisks) are found distributed in the epidermis. Reflective elements (arrows) are identified as round granules in the dermal layer close to chromatophores (arrowheads). **(F)** Bright-field (BF) imaging of the same section counterstained with DAPI showed chromatophores (arrowhead) and reflective elements (arrow) underneath the epidermal layer, where mucous cells (asterisk) are identified based on morpholgy and position. Scale bars: 150 µm in **(B,C)**, 50 µm in **(D,E)**, and 20 µm in **(F)**. Abbreviations: ch, chromatophores; ep, epidermis; mv, microvilli; ref, reflective elements.

In the skin covering the arm (Figure 1A), chromatophores were easily identified as bright green spots (TPEF) distributed over the entire arm structure, which, in turn, appears in red (CARS) in multichannel images (Figures 1B,C, whole mount sample).

Higher-magnification imaging of sagittal thin sections of the arm allowed for identification of other distinctive elements of the skin and surrounding tissues. The epidermis appeared covered in microvilli, characterized by an intense CARS signal; mucous cells were identified as negative imprints in the epidermal layer (Figures 1D–F, asterisks); and close to chromatophores (Figures 1D–F, arrowheads), reflective elements appeared as round granules just underneath the epidermis (CARS, Figures 1D–F, arrows). Bright-field imaging (i.e., transmitted white light) of the same section highlighted the nuclear components of the epidermis (DAPI counterstain in blue in Figure 1F), confirming the identity of mucous cells by morphology and nuclear position.

**FIGURE 2:**
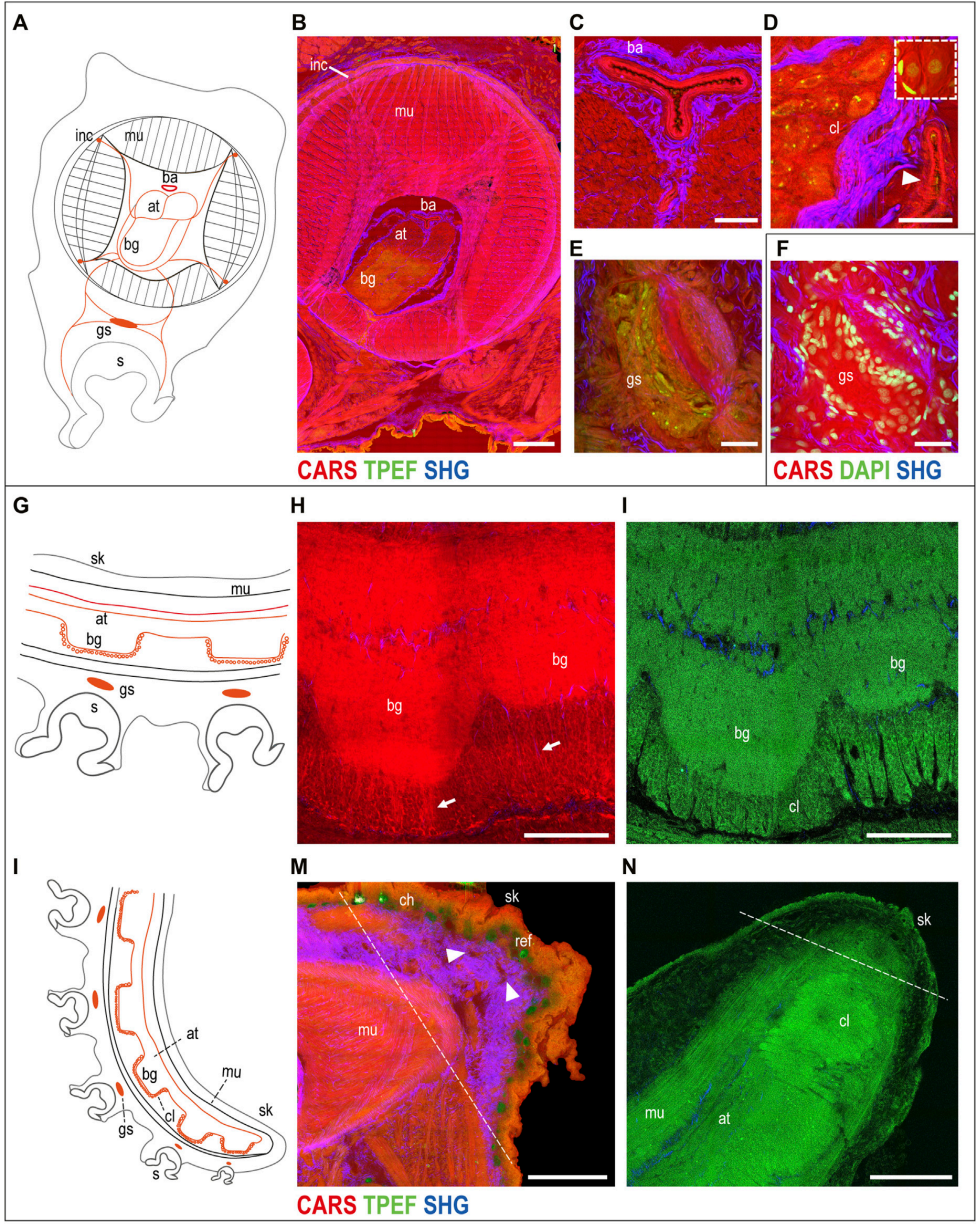
Uninjured arm. Schematic drawings of octopus arm morphology in transverse **(A)** and sagittal **(G,L)** planes. **(B)** Multiphoton microscopy image of an arm transverse section showing the three muscle bundles belonging to the intrinsic musculature of the arm (CARS) and the connective tissue sheaths enveloping them (SHG). The axial nerve cord (comprising two axonal tracts on the dorsal side and brachial ganglia on the ventral side) and the four intramuscular nerve cords are clearly identified (CARS and TPEF). **(C)** Above the axonal tracts, the brachial artery is visible (CARS) surrounded by connective tissue (SHG). **(D)** Outer cellular layer of a brachial ganglion appears comprising small and big neurons emitted in CARS and TPEF. DAPI counterstaining (white dotted rectangle) highlights neuron nuclei and supporting cells surrounding them. The arrowhead points a blood vessel around the nerve cord. **(E)** Ganglion of the suckers comprising a central neuropil and surrounding neurons. **(F)** Neuron nuclei are counterstained in DAPI for further confirmation. **(H,I)** Imaging of the axial nerve cord in the thick sagittal section (150 µm). **(H)** Fibers from the brachial ganglia descend from the neuropil (arrows), passing through the **(I)** cellular layer. **(M)** Most distal part of the arm tip (delimited by a dotted line) presents numerous blood vessels (arrowheads). **(N)** Single plane from arm tip whole-mount imaging (in TPEF). Dotted line delimits the most distal part of the arm tip. Scale bars: 500 µm in **(B)**, 100 µm in **(C,E,F,H,I)**, 50 µm in **(D)**, and 200 µm in **(M,N)**. Abbreviations: at, axonal tract; ba, brachial artery; bg, brachial ganglion; ch, chromatophores; cl, cellular layer; gs, sucker ganglion; inc, intramuscular nerve cord; mu, muscles; ref, reflective elements; s, sucker; sk, skin.

**The muscular tissue.** A schematic drawing of the arm morphology in the transverse plane is included in Figure 2A to facilitate structural identification (Supplementary Figure S2A). The three muscle groups belonging to the intrinsic musculature of the arm were visualized in CARS (Figure 2B). In particular, the i) tightly packed transverse muscle bundles running perpendicular to the arm long axis, which elongate through structures called trabeculae, were visible among the ii) longitudinal muscles and the iii) three bundles of oblique muscles (see Supplementary Figure S2 for more details). Connective tissue sheaths, highlighted in SHG, appear to envelop the different muscle layers of the intrinsic musculature (Figure 2B).

CARS imaging also enabled identification of the intrinsic musculature of the sucker (data not shown) and the acetabulo-brachial muscles (Supplementary Figure S2), which connect the intrinsic muscles of the arm and the intrinsic muscles of the suckers.

**The neural structures.** The neural control for these sets of muscles is provided by six main nerve centers per arm, that is, a central axial nerve cord connected to four intramuscular nerve cords and to sucker ganglia. The axial nerve cord comprises two axonal tracts (dorsal) and several brachial ganglia (ventral), facing and innervating suckers (Figures 2A,B; Supplementary Figure S2). Running longitudinally to the axonal tracts, the main blood vessel supplying hemolymph to the arm (brachial artery) is shown by CARS and is surrounded by connective tissue (SHG) (Figures 2B,C; Supplementary Figure S2B).

Each brachial ganglion comprises an inner neuropil and an outer cellular layer (Figures 2B,D; Supplementary Figures S2B–D). The cellular layer contains many small and some big neurons, with nuclei ranging from less than 5–20 µm (Young 1963). These cells emit both in CARS and TPEF, with the latter mainly highlighting their cytoplasm, giving a strong signal of granular structures contained in the cells (Figure 2D; Supplementary Figure S2C) that could be partly due to lipofuscin. DAPI counterstaining confirmed these results and highlighted the presence of supporting cells around the neurons, which were not detected with multiphoton microscopy alone (see the dotted white rectangle in Figure 2D **)**. Axons in the intricate neuropil of the brachial ganglia are also highlighted in CARS and TPEF (Figure 2B; Supplementary Figures S2B,C). DAPI counterstaining and acetylated tubulin immunoreactivity confirmed these results (Supplementary Figure S2D).

Some of the nerves departing from the brachial ganglia are linked to the four intramuscular nerve cords (CARS; Figure 2B; Supplementary Figure S2B), and to the ganglion of the sucker (CARS and TPEF; Figures 2E,F). This ganglion comprises a central neuropil and neurons surrounding it (TPEF and DAPI) (Figures 2E,F).

Imaging of the arm tip allowed visualization of the abovementioned anatomical structures (a schematic drawing in the sagittal section is reported to facilitate structure identification, Figures 2G,L).

Compared to more aboral arm portions, in the tip, we observed a greater area occupied by the axial nerve cord, reducing the space for muscles; brachial ganglia get closer to each other (Figures 2H,I,N) as suckers get smaller and closer. Fibers from the brachial ganglia descend from the neuropil (CARS, Figure 2H, arrow), passing through the cellular layer (TPEF, Figure 2I).

Tissues and structures at the most distal part of the tip (delimited by a dotted line in Figures 2M,N) appear less organized and differentiated compared to all the other neighboring areas (Supplementary Figure S3). The tip appears characterized by a thick layer of connective tissue (SHG) (Figure 2M), which appears in between the epidermis and the muscular layer covering the nerve cord. CARS also highlighted the presence of numerous blood vessels in this zone (Figure 2M, arrowhead).

### Healing Arm

**FIGURE 3:**
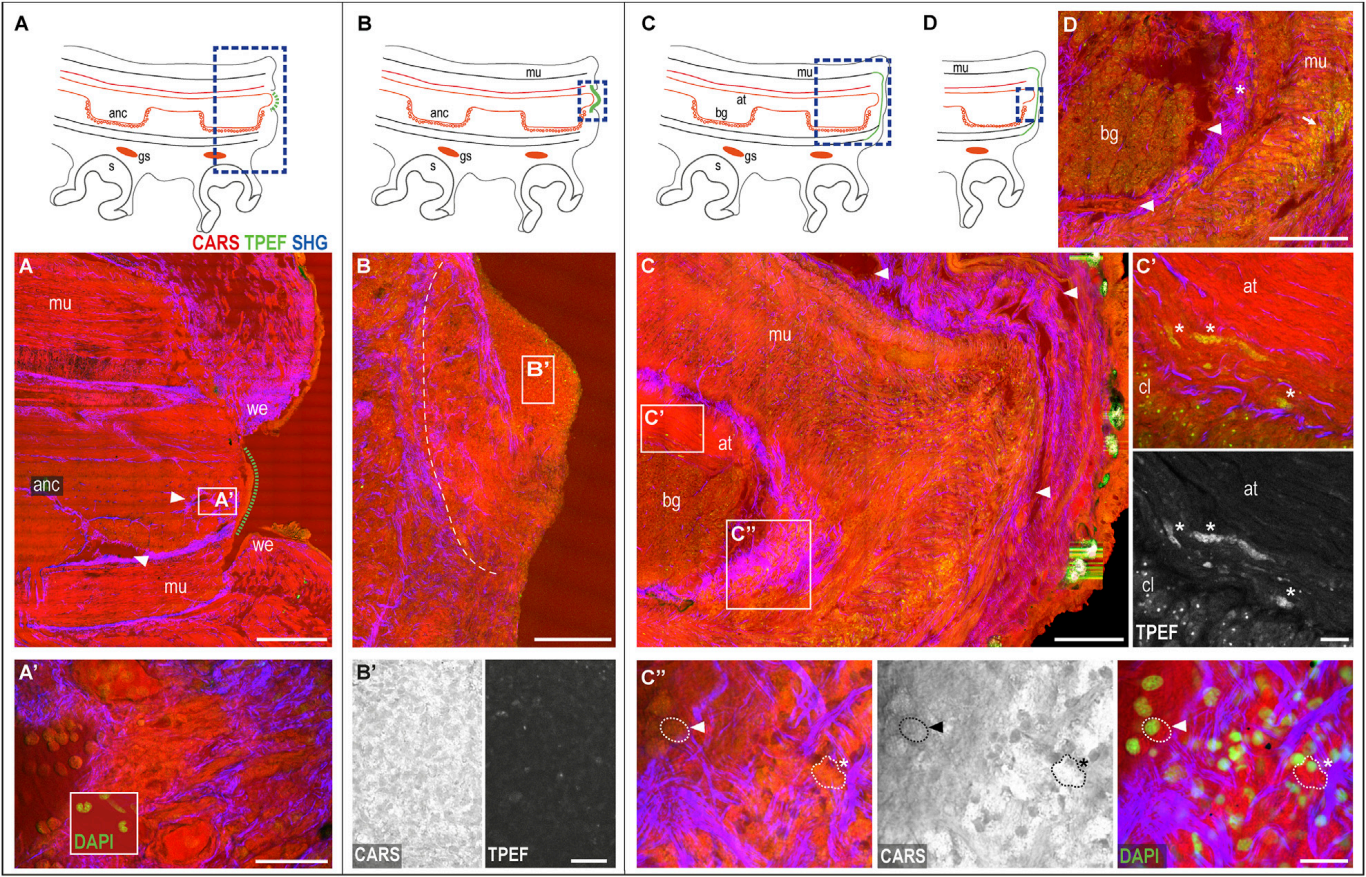
Phases of the arm healing process. Schematic drawings of an arm in the sagittal section **(A–D)** Describing healing phases imaged with multiphoton microscopy. **(A)** Wounded dermis forms a rim contracting around the wound. The connective tissue (SHG) narrows around the muscular tissues of the stump contributing to wound closure. A clot of agglutinated blood cells deposits over it (green dotted line) originating from blood vessels in the stump (arrowheads). **(A′)** Hemocytes released in the stump. DAPI counterstaining shows the peculiar u-shaped nucleus of the hemocytes, which occupies most of the cytoplasm. **(B)** Clot covers the whole exposed tissue in between the wound epithelium. A boundary line (white dotted line) of the connective tissue (SHG) separates the clot from the underlying and well-differentiated tissues of the stump. **(B′)** Clot appears as a dense and fine network of interdigitated cells, **(C)** Regenerating epithelium and highly vascularized (arrowheads) covers the clot. Damaged muscles and axonal tracts degenerate **(C′)** (asterisks). **(C′′)** Hemocytes are released from the blood stream. They change their appearance from circulating round-shaped cells with a small cytoplasm and u-shaped nuclei (dotted line with arrowhead) into amebocyte-like cells with a large and granular cytoplasm, intensely emitted in CARS and TPEF (dotted line with asterisks). DAPI counterstaining shows the nuclei of these cells. **(D)** Hemocytes are released from the vessels around the nerve cord (arrowheads) into the connective tissue around it (asterisk), then, invade all muscle layers below the wounded epithelium (arrow). Scale bars: 500 µm in **(A)**, 50 µm in **(A′)**, 20 µm enlargement in **(A′)**, 200 µm in **(B–D)**, and 20 µm in **(B′,C′,C′′)**. Abbreviations: at, axonal tract; bg, brachial ganglion; cl, cellular layer; gs, sucker ganglion; s, sucker; we, wound epithelium.

**The wounded skin.** The regenerative process of a damaged arm in *O. vulgaris* is always initiated by wound healing, with the dermis wound edges closing around the lesion. This process generally requires between 0 and 5 days, depending on several factors, such as temperature, animal age and sexual maturity, and health status (for a review, see Imperadore and Fiorito, 2018). To facilitate readers, the main phases of the healing process are sketched in Figures 3A–D.

The wounded dermis contracts and forms a rim that starts covering the wound to form a first protective layer for the exposed tissues (Figure 3A). The connective tissue within it appears involved in the process, narrowing around the muscular tissues of the stump (SHG, Figure 3A) and contributing to wound closure.

The central portion of the damaged arm (i.e., the internal muscles and axial nerve cord) remains exposed until a clot of agglutinated blood corpuscles start depositing over it (green dotted line in Figure 3A). Blood vessels, which are observed in great number in the arm stump (Figure 3A, arrowheads), represent the origin of these cells (Figures 3A′, see also inset in Figure 3A′).

The clot then increases in size, covering the whole exposed tissue in between the wound epithelium (Figure 3B) and forming a dense and fine network of interdigitated cells called primary blastema (Lange, 1920). The cells in this blastema appear full of dense granules highlighted in CARS and TPEF (Figure 3B′). A boundary line of connective tissue separates this blastema from the underlying and well-differentiated tissues (SHG, Figure 3B). The primary blastema finally never casts off, but rather is retained and eventually completely covered by the regenerating epithelium, the latter appearing highly vascularized (Figure 3C, arrowheads).

**The muscular and neural tissues.** At this stage, the damaged muscles and nerve tissues (i.e., the axonal tracts) show evident signs of degeneration (i.e., swelling and fragmentation). Degenerating tissues, highlighted in CARS and TPEF (Figure 3C′, asterisks) are not observed in control tissues.

**The hemocytes.** The muscular layers in the healed stump are invaded by many cells (Figure 3C), identified as hemocytes, which change their appearance once released from blood vessels. They indeed transform from circulating, round-shaped cells with a small cytoplasm and u-shaped nuclei (Figure 3C′′, dotted line with arrowhead) into amebocyte-like cells with a large and granular cytoplasm, intensely emitting in CARS and TPEF (Figure 3C′′, dotted line with asterisk). DAPI counterstaining confirmed the cellular nature of these structures (Figure 3C′′). Hemocytes released from the vessels around the nerve cord (Figure 3D, arrowheads) are first released into the connective tissue around it (Figure 3D, asterisk) and then invade all muscle layers below the wounded epithelium (Figure 3D, arrow).

### Regenerating Arm

**FIGURE 4:**
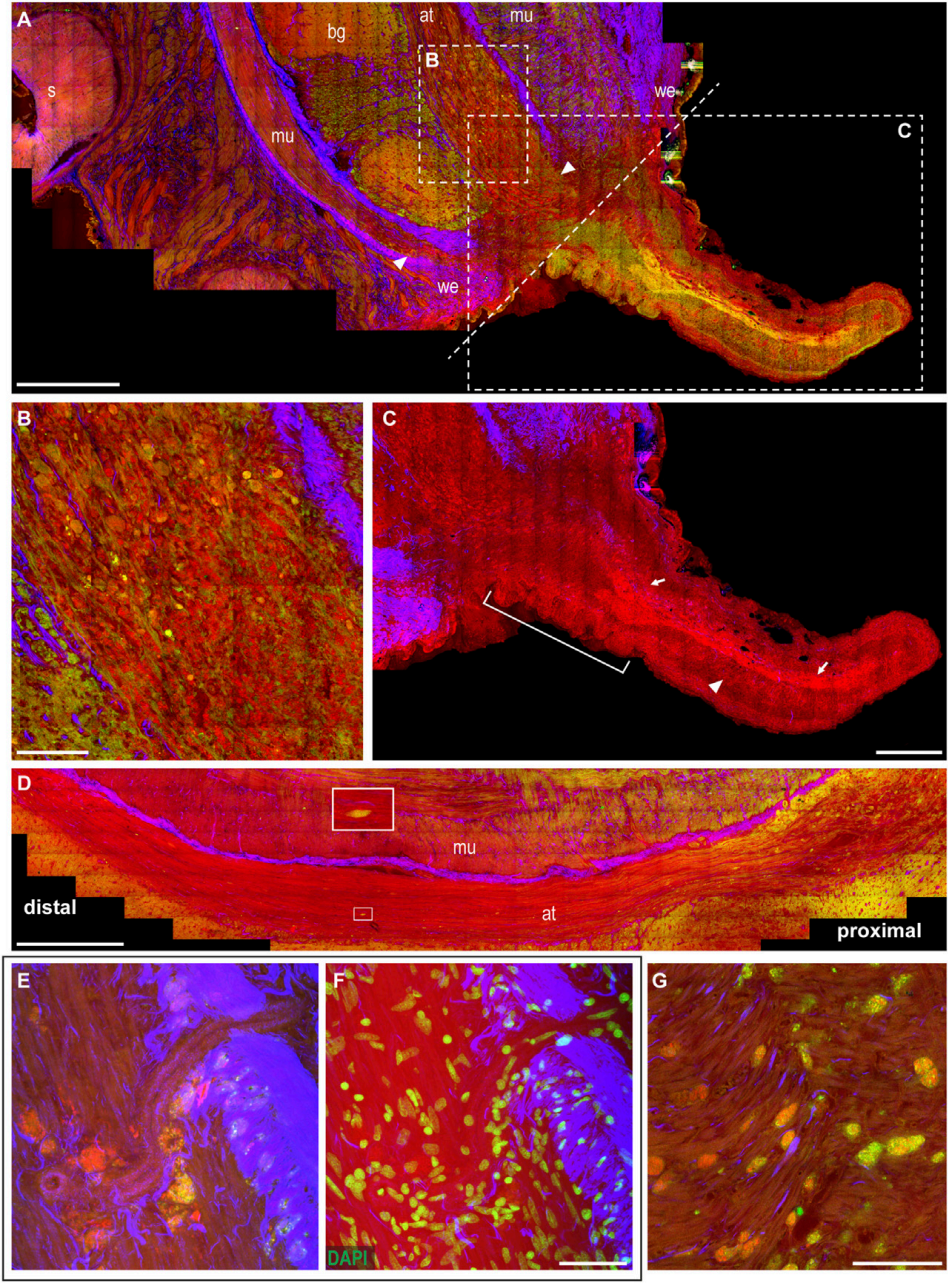
Arm regeneration. **(A)** Little knob regenerates from the dorsal side of the stump. The wound epithelium narrows around the original site of the lesion, characterized by thick connective tissue. White dotted line marks the original site of the lesion dividing the stump from the regenerating tip. **(B)** Degeneration is evident in the axonal tracts of the nerve cord, where fibers appear swollen and break into lumps (CARS and TPEF). **(C)** Regenerating fibers appear among degenerating lumps in the axonal tract (highlighted in CARS) and can be followed providing innervation in the newly forming tip (arrows); thin processes can be seen descending perpendicularly to these fibers (arrowhead). Suckers develop at the base of the regenerating arm (white bar). **(D)** Degeneration can be followed along the axonal tracts: it involves a great number of fibers proximal to the lesion; few degenerating fibers can be detected moving distally (see enlargement). **(E,F)** Large number of cells, whose cytoplasm is rich in granules emitting in CARS and TPEF, are imaged accumulating close to blood vessel walls. DAPI counterstaining highlighted cell nuclei. **(G)** Cells rich in granules emitted in CARS and TPEF invade muscle layers in the stump. Scale bars: 500 µm in **(A,D)**, 100 µm in **(B,G)**, 250 µm in **(C)**, and 50 µm in **(E,F)**. Abbreviations: at, axonal tract; bg, brachial ganglion; mu, muscles; s, sucker; we, wound epithelium.

**The wounded skin.** From the healed skin, a little knob appears, regenerating an arm from the dorsal side of the stump. The resulting arm is initially much thinner than the original stump (Figure 4A). The wound epithelium, narrowing around the original site of the lesion, is still visible and characterized by thick connective tissue (SHG, Figures 4A,C).

**The muscular and neural tissues.** Degeneration in this phase involves greater areas in the muscular tissues and the nerve cord of the stump (Figure 4A). Degeneration is particularly evident in the axonal tracts of the nerve cord, close to the original site of the lesion, where fibers appear swollen and broken into lumps (CARS and TPEF, Figure 4B). Degeneration can be followed along the axonal tract, with the number of fibers involved decreasing when farther from the site of the lesion. Distal to this site, fewer degenerative events are found using multiphoton imaging (Figure 4D).

Among the degenerating lumps in the axonal tract, regenerating fibers also appear (highlighted by strong CARS signal, Figure 4B) and can be followed providing innervation in the newly forming arm tip (Figure 4C, arrows). The regenerating tip is mostly occupied by the newly forming nerve cord (Figure 4C, arrows), and thin processes can be seen descending from it toward the ventral site, where new suckers will later form (Figure 4C, arrowhead). Suckers start to develop in the forming arm, close to the stump (Figure 4C).

**The hemocytes.** The tissues in the stump are invaded by cells whose cytoplasm is rich in small granules, strongly emitting in CARS and TPEF (Figures 4E–G). They mainly invade muscles around the nerve cord and the axonal tracts of the latter (see also Supplementary Figure S4A, arrowheads), but are never observed in the neuropil of the brachial ganglia. These cells also reach muscles in the regenerating arm tip, very close to the site of the lesion (Supplementary Figure S4A), but are not found distant to this site or in any other tissue of this new structure.

These cells appear to be released by blood vessels close to the injury site (Supplementary Video S1).

Areas invaded by these structures are also characterized by numerous proliferating cells (Supplementary Figure S4B).

## Discussion

Multiphoton imaging has been successfully applied to several species to investigate a number of biological processes (Zipfel et al., 2003) including healing and regeneration.

Multimodal images (CARS, TPEF, and SHG) of *O. vulgaris* uninjured and damaged arms allowed for the identification of the cellular and structural elements characterizing the parts and contributing to appendage regeneration, helping in dissecting this complex phenomenon in the absence of specific markers available for the taxon. In particular, chromatophores—skin element key for body patterning—and muscular bundles—contributing to motor patterns of the arm and the main neural components—were detected (Figures 1, 2; Supplementary Figure S2).

Wound healing is a phenomenon with widespread occurrence among both vertebrates (e.g., *Ambystoma mexicanum* and *Danio rerio*) and invertebrates (e.g., *Caenorhabditis elegans* and *Drosophila melanogaster*), also occurring in mammals. This involves the activation of the immune response and the remodeling of the extracellular matrix (Arenas Gómez et al., 2020), with regenerative species sharing impressive similarities in the process.

In octopus, healing is marked by dermis contraction, which eventually covers the clot of agglutinated corpuscles depositing over the exposed tissue to form the blastema. Hemocytes invade the stump, changing their appearances from circulating, round-shaped cells to amebocyte-like cells (Figure 3). These latter cells resemble vertebrate macrophages (Aurora and Olson, 2014; Uckermann et al., 2019), thus suggesting their involvement in debris removal.

After complete healing, a little tip regenerates from the octopus arm stump with new fibers innervating it. Cells rich in small granules (CARS and TPEF), likely hemocytes, are found to invade muscles and nerve tissues which are also characterized by intense proliferation (Figure 4; Supplementary Figure S4).

Here, we imaged structures and cells involved in arm regeneration in the octopus, bypassing the need for staining or markers, enabling the collection of voluminous data in a short period of time. Additionally, scanned samples are suitable for further processing, for instance IHC and staining, allowing for amplified saving of time and resources and reducing the number of samples and experimental animals needed, thereby contributing to better compliance with the 3R Principle (Fiorito et al., 2014; https://nc3rs.org.uk/the-3rs).

This approach could be extended to other lines of cephalopod research and to different non-mammalian animal species, enabling data collection without having to focus on one or a few proteins, as is usually the case with IHC approaches.

## Data Availability

The original contributions presented in the study are included in the article/Supplementary Material, further inquiries can be directed to the corresponding author.
