# Supplementary material for: Imaging Arm Regeneration: Label-Free Multiphoton Microscopy to Dissect the Process in Octopus vulgaris
Source: Front Cell Dev Biol. 2022 Feb 4;10:814746. doi: 10.3389/fcell.2022.814746 (PMC8855035; doi:10.3389/fcell.2022.814746)
Supplement: Supplementary file 2 [file DataSheet1.docx]

**
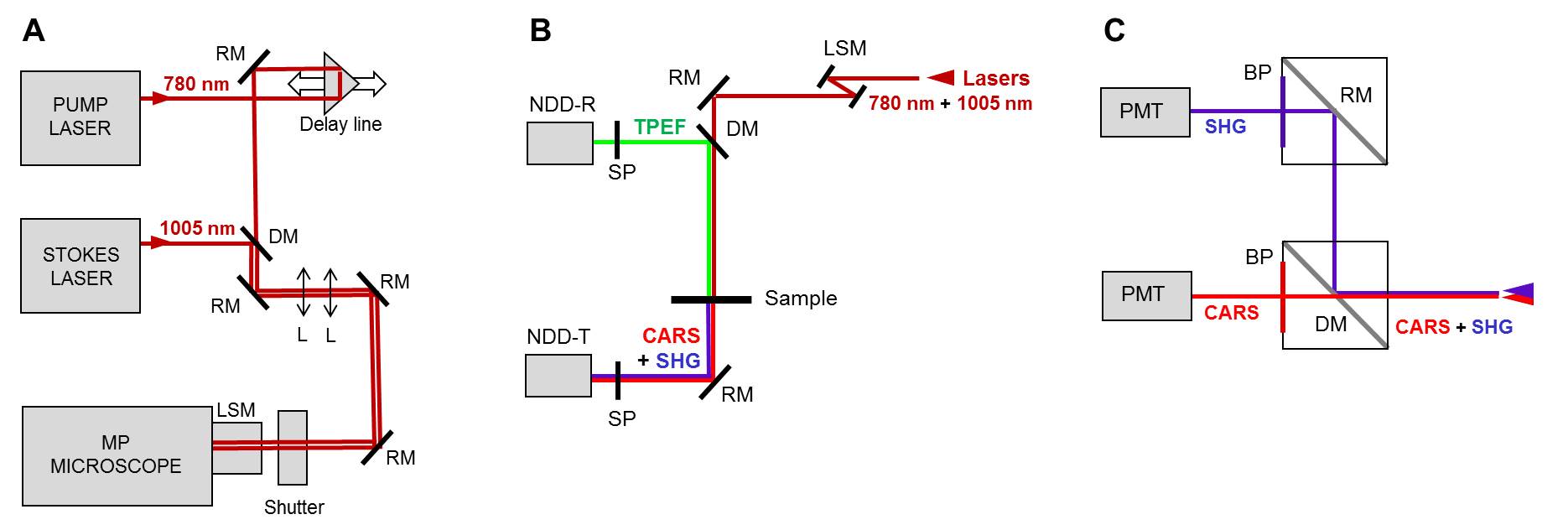
**

**Supplementary Figure 1: Diagram of the system for multiphoton microscopy.**

(A) Diagram of the whole system, consisting of pump and Stokes pulsed lasers with emission at 780 nm and 1005 nm respectively, delay line to overlap the pulses in the time domain and dichroic mirror (DM) acting as beam combiner. Reflective mirrors (RM) are used for beam steering and two lenses (L) are used to recollimate the beams in order to obtain the correct beam diameter the entrance of the laser scanning module (LSM) of the multiphoton microscope. A shutter enables laser emission only during the measurement to guarantee the operator safety.

(B) Diagram of the multiphon microscope, with the laser scanning system (LSM) for beam rastering and reflecting mirrors (RM) to bring the laser beam to the sample. The TPEF signal is acqured in reflection configuration, being reflected towords the reflection non-descanned detection unit (NDD-R) by a dichroic mirror (DM). CARS and SHG signals are preferentially emitted in forword direction and are therefore acquired in transmission configuration by a two-channel non-descanned detection unit (NDD-T). Short-pass filters (SP) prevent the backscattered and forward transmitted laser radiation from entering the detection units.

(C) Diagram of the two-channel non-descanned detection unit (NDD-T) for CARS and SHG detection, consisting in two filter cubes with a dichroic (DM) or reflecting mirror (RF) and band-pass filters (BP) to split CARS and SHG signals exploiting the different wavelength, and two photomultiplier tubes (PMT) for signal acquisition.

**
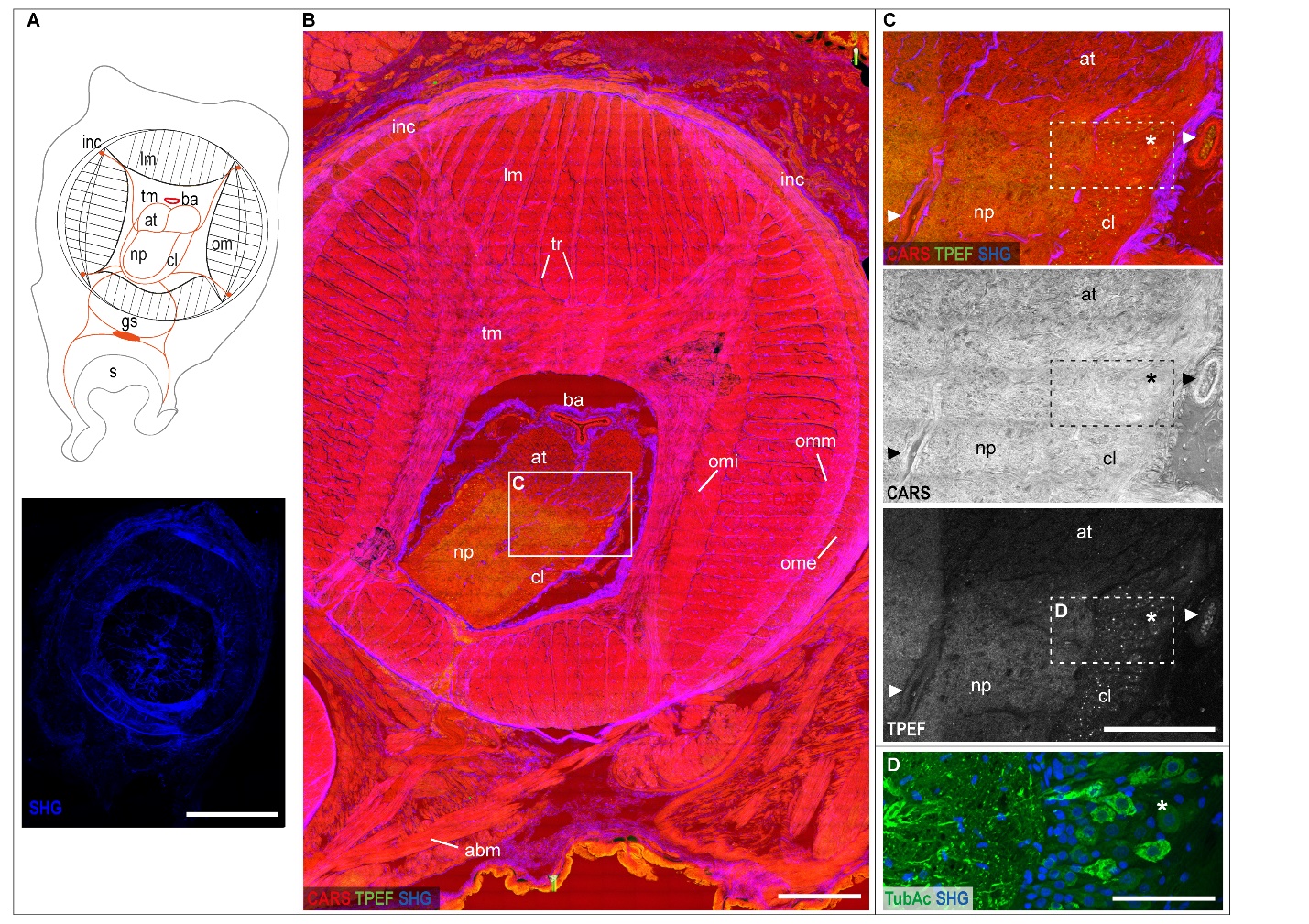
**

**Supplementary Figure 2. Uninjured arm**

(A) Schematic drawing of *O. vulgaris* arm morphology (top) and SHG imaging (bottom) of transverse section.

(B) Multiphoton microscopy image of an arm transverse section showing muscle layers of the intrinsic musculature of the arm (CARS) and the connective tissue sheaths enveloping them (SHG).

(C) Enlargement of the axial nerve cord as multimodal image (top) and single channel (CARS and TPEF) allows identification of axonal tract and brachial ganglion, the latter composed of an outer cellular layer and a central neuropil.

(D) The same slice underwent IHC protocol with TubAc antibody and DAPI counterstain, detecting fibers in the neuropil and neurons in the cellular layer (asterisk).

**Scale bars:** 500 µm in A (SHG) and B, 200 µm in C, 100 µm in D.

**Abbreviations:** at, axonal tract; ba, brachial artery; cl, cellular layer; gs, sucker ganglion; inc, intramusclular nerve cord; lm, longitudinal muscles; np, neuropil; om, oblique muscles; ome, external oblique muscle layer; omi, internal oblique muscle layer; omm, median oblique muscle layer; s, sucker; tm, transverse muscles; tr, trabeculae.


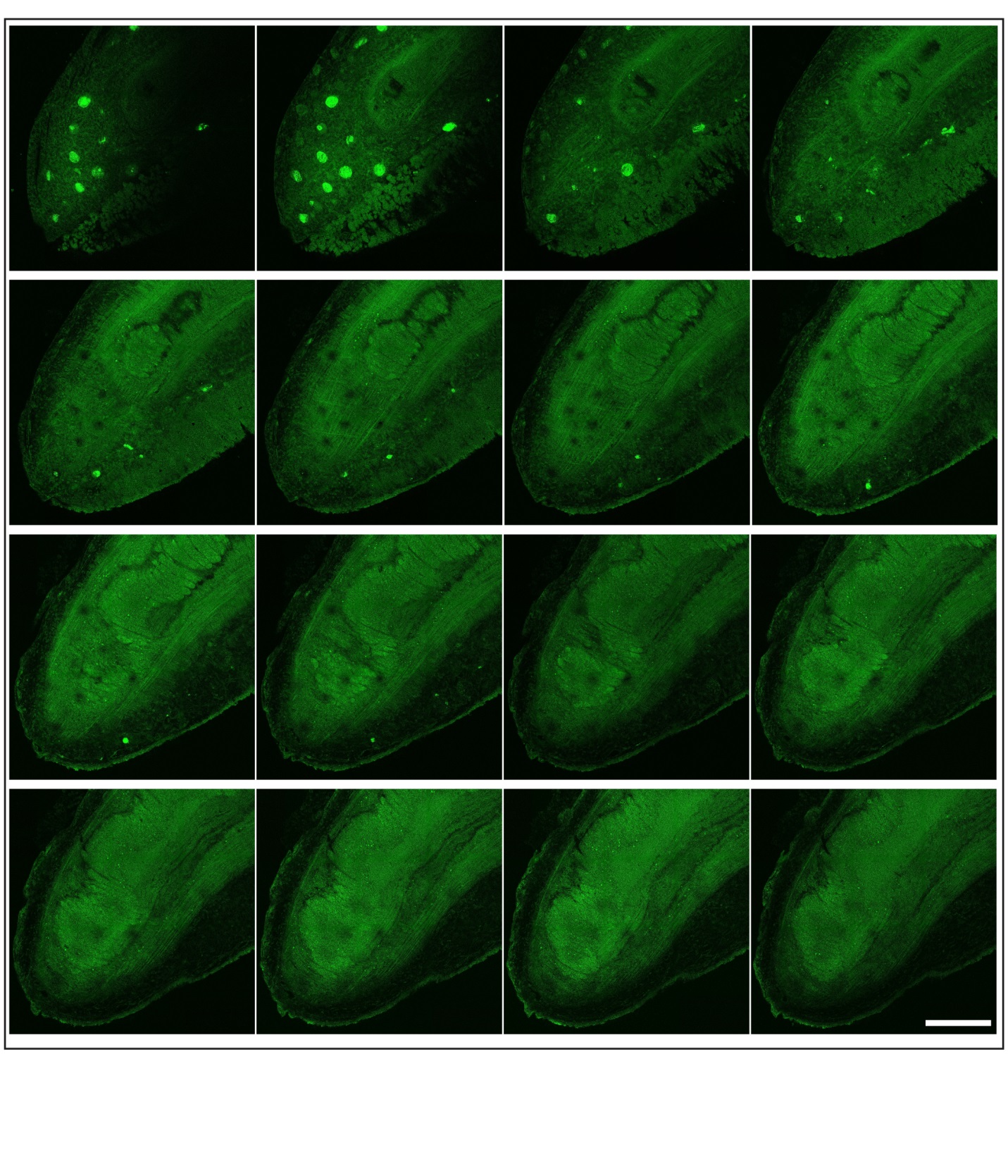


**Supplementary Figure 3. Whole mount tip imaging.**

Z-stack imaging of a whole mount tip (TPEF, green) starting from skin. Voxel size of 0.2 µm x 0.2 µm x 3.0 µm, 16 out of 36 frames shown.

**Scale bar:** 250 µm


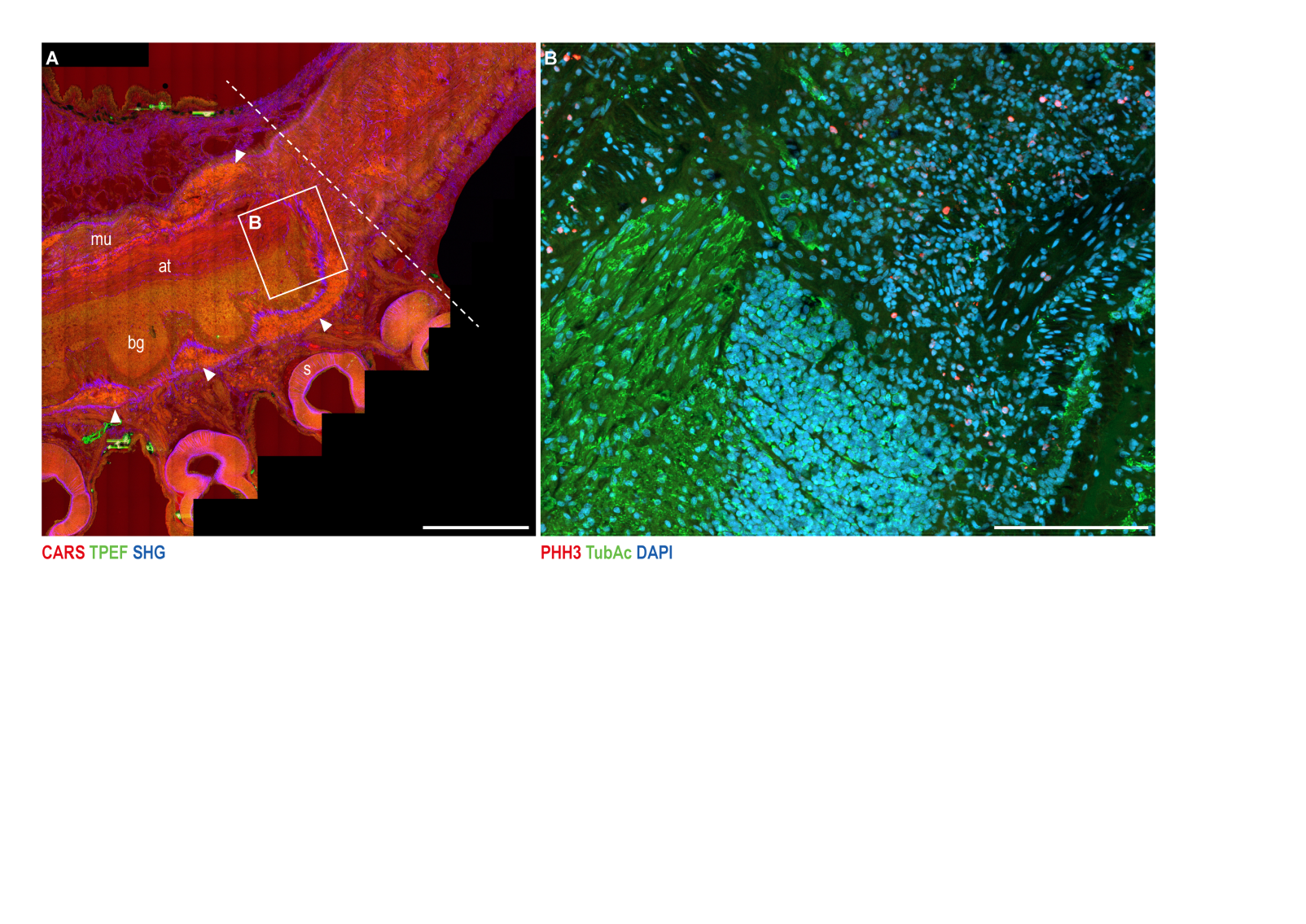


**Supplementary Figure 4. Invading cells.**

(A) Invading cells accumulating around the nerve cord of the stump (arrowheads). White dotted line marks the original site of lesion, dividing the stump from the regenerating tip.

(B) Areas invaded by granulated cells is characterized by numerous mitotic cells (PHH3)

**Scale bars:** 500 µm in A, 150 µm in B.

**Abbreviations:** at, axonal tract; bg, brachial ganglion; mu, muscles; s, sucker.

**Supplementary Video 1. Hemocytes release**

Hemocytes in blood vessels released into the injury site
